# Computational Models of Claudin Assembly in Tight Junctions and Strand Properties

**DOI:** 10.3390/ijms25063364

**Published:** 2024-03-16

**Authors:** Sarah McGuinness, Samaneh Sajjadi, Christopher R. Weber, Fatemeh Khalili-Araghi

**Affiliations:** 1 Department of Biomedical Engineering, University of Illinois Chicago, Chicago, IL 60607, USA; smcgui5@uic.edu; 2 Department of Mechanical Engineering, University of Illinois Chicago, Chicago, IL 60607, USA; ssajja6@uic.edu; 3 Department of Pathology, University of Chicago, Chicago, IL 60637, USA; cweber@bsd.uchicago.edu; 4 Department of Physics, University of Illinois Chicago, Chicago, IL 60607, USA

**Keywords:** tight junction, claudin, molecular dynamics, ion channel, ion transport, claudin assembly

## Abstract

Claudins are one of the major components of tight junctions (TJs) that polymerize within the cell membrane and form interactions between cells. Some claudins seal the paracellular space, limiting paracellular flux, while others form selectively permeable ion channels that control the paracellular permeability of small ions. Claudin strands are known to be dynamic and reshape within TJs to accommodate large-scale movements and rearrangements of epithelial tissues. Here, we summarize the recent computational and modeling studies on claudin assembly into tetrameric ion channels and their polymerization into μm long strands within the membrane. Computational studies ranging from all-atom molecular dynamics, coarse-grained simulations, and hybrid-resolution simulations elucidate the molecular nature of claudin assembly and function and provide a framework that describes the lateral flexibility of claudin strands.

## 1. Introduction

Claudins (cldns) are cell–cell adhesion proteins that form the backbone of tight junctions (TJs) and selectively seal the paracellular spaces in epithelia [1,2,3,4,5]. Cldns polymerize in the cell membrane and form a network of strand-like structures that connect to a similar network on neighboring cells [5,6]. They form a barrier to control the transport of small molecules across epithelia [7,8,9,10,11,12]. Dysregulated cldn expression or function has been identified in various cancers, kidney diseases, and intestinal diseases, underscoring the therapeutic potential for targeting claudins [13,14].

So far, 27 members (subtypes) of the cldn family have been identified [8]. Beyond determining the barrier or channel properties, the composition of cldn subtypes is observed to influence the morphologies of the TJs and cldn strands [4,7,10,15,16,17,18,19,20,21,22,23,24]. TJ strands are dynamic and respond to physiological forces by continuously reshaping and forming new branches [25,26,27,28]. However, it remains unclear how cldn strands within TJs bend, arch, or form new branches without losing their barrier function. Here, we present an overview of the recent computational studies on cldn strand assembly and their contribution to our understanding of barrier function. In particular, we will discuss the mechanical properties of cldn strands and the molecular mechanism for the lateral flexibility of cldn strands in the membrane. 

## 2. Cldn-15: From Crystal Structure to Computational Model

**Figure 1:**
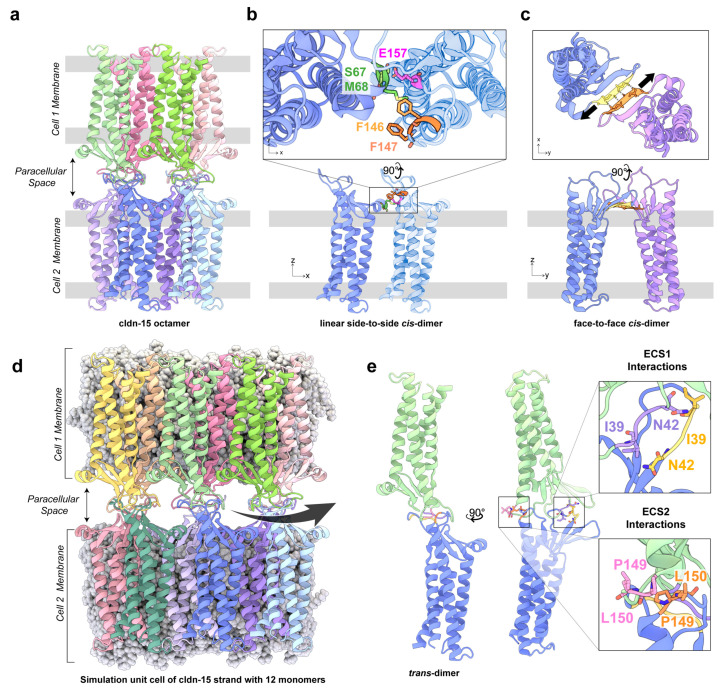
(**a**) Cldn channels are primarily composed of 4 monomers (purple, blue, green, and pink), with extracellular loops of 4 additional neighboring monomers (light pink, light green, light blue, and light purple) contributing to the paracellular channel walls, as shown by the cldn-15 octamer. Cldns polymerize via three distinct sets of interactions. For claudins in the same membrane, they participate in (**b**) side-to-side *cis* interactions, in which E157, F146, and/or F147 on one cldn interact with S67 and M68 on the ECH of a neighboring cldn; (**c**) face-to-face cis interactions involving hydrogen bonds between the backbone atoms of residues 60–64 on β4 of two neighboring cldns; (**d**) between cldns in opposing membranes via head-to-head *trans* interactions, as shown in the simulation unit cell of a cldn-15 strand composed of 12 monomers [33]; and (**e**) head-to-head *trans* interactions include hydrogen bonds between ECS1 residues 39 to 42 and ECS2 residues 149, 150.

The crystal structure of cldn-15 was determined in 2014, revealing a left-handed bundle of four transmembrane helices (TM1–TM4), an extracellular domain with a five-strand β-sheet (β1–β5), and two extracellular loops [29]. Cysteine crosslinking experiments and freeze-fracture electron microscopy observations of TJ strands led to the proposal of the Suzuki model for cldn assembly in TJs [30]. In this model, cldns polymerize into an antiparallel, double-row strand in a membrane (*cis* polymerization) that is then connected to a similar double-row structure on an opposing membrane (*trans* polymerization) [30]. The lateral arrangement of cldns in a single row was adapted from the crystallographic packing of cldn-15 (side-by-side *cis* interactions) and involved an interaction between the extracellular helix (ECH) of one protomer and the extracellular loop ESC2 (residues F146, F147, and L158) of the adjacent protomer (Figure 1a,b). In addition to side-to-side *cis* interactions, the Suzuki model suggested a “face-to-face” *cis* interaction between two antiparallel β-sheets (β5-β5) that was critical for the double-row arrangement (Figure 1c). The *trans* interactions mainly involved the extracellular loops of cldns (Figure 1d,e), which were not fully resolved in the crystal structure, but were suggested to play a key role in cldn assembly into paracellular pores [30,31,32,33]. Cldn polymerization in this model enables the formation of ion channels that run parallel to the two membranes, defining a paracellular pathway for the transport of small ions. In this arrangement, the pore’s backbone is composed of a β-barrel formed by the extracellular domains of four monomers.

The elucidation of the cldn-15 structure provided the impetus to assemble the first in silico prediction of the cldn-15 multimeric structure, with the addition of extracellular residues 31–41 between β1 and β2, which are missing from the crystal structure, and models of the *trans* interactions [33,34,35]. These molecular dynamics studies verified the stability of the Suzuki model in two parallel lipid membranes and demonstrated the roles of side-by-side and face-to-face interactions in stabilizing the three-dimensional structure of the pore. Furthermore, they demonstrated that cldn-15 in this arrangement forms cation-selective channels with a well-defined selectivity filter centered around four D55 residues in the middle of the pore [33,34]. In addition, Samanta et al. [33] verified the model against in vitro electrophysiological studies of cldn-15 and its mutants, concluding that the pore surface captures key residues that control the charge selectivity and size selectivity of cldn-15 [33,36,37]. 

More recently, the Suzuki model was applied to cldn-10b, a pore-forming cldn with the highest sequence similarity to cldn-15 [38]. The authors compared two variants of the Suzuki model with subtle differences in the orientation of the ~10 amino acid β1β2 loop of the extracellular domain. This subtle variation led to the emergence of two distinct pore structures: a tetrameric barrel conformation, with the β1β2 loop oriented toward monomers in the opposing membrane, similar to the cldn-15 models of Alberini et al. [34,35]; and an octameric interlocked-barrel conformation, in which the β1β2 loops extend towards monomers in the same membrane and protrude into the adjacent pores, similar to the cldn-15 models of Samanta et al. [33] and McGuinness et al. [37]. The latter indicates that each pore is formed by the confluence of eight monomers rather than four, with β-sheets from the four monomers constituting the bulk of the pore’s structure, while β1β2 loops from the four neighboring monomers also contribute to the pore walls. An extensive analysis of these two conformational model variants revealed the octameric interlocked-barrel conformation as more stable, exhibiting more robust face-to-face *cis* interfaces and ECS2–ECS2 *trans* interfaces than the tetrameric locked-barrel conformation [38]. In the next section, we will discuss possible cldn–cldn *cis* interfaces and their functional implications in classic cldns. 

## 3. Multiple Cldn–Cldn *cis* Interfaces

The morphology of TJ strands, i.e., the number of strands, their branching, and the continuity of the strand, varies among different tissues and is assumed to depend on cldn subtypes [12,17,19,20]. Live cell imaging of cldn-2 strands indicates that cldn strands are highly flexible and capable of fluctuating between straight and curved morphologies [39]. Freeze-fracture electron microscopy of TJ strands formed by cldn-15 show a wide range of curvatures that suggest a high lateral flexibility of TJ strands in the membrane [40,41]. The lateral flexibility of the strands is mainly attributed to multiple *cis* interfaces among cldn monomers. Zhao et al. used a combination of docking, sequence analyses, and molecular dynamics simulations to investigate alternative *cis* interactions between cldn-15 monomers [39]. They identified an alternative *cis* interface between cldn-15 monomers corresponding to a 17° rotation of the main interface in the crystal packing [30]. This new interface, while very similar to the original interface, contained new interactions mainly between ECS2 (E157) and ECH (S67) or R79. Although the experimental structure initially suggested the roles of S67 and E157 in strand formation [29], electron microscopy images of cldn-15 mutants revealed that strand formation is still possible when S67 and E157 are mutated. However, the ultrastructure of the mutant strands was strongly altered, showing discontinuous strands with a reduced persistence length [39]. These results indicate that TJs tolerate modifications of their main interaction site, consistent with the hypothesis that cldn polymerization is reinforced through multiple interactions or interfaces.

CG simulations of cldn-5 in lipid membranes demonstrated the self-assembly of cldn-5 into dimers with multiple interfaces [42,43,44]. The authors suggested that these dimeric interactions could lead to *trans* interactions and proposed two putative models for cldn pores (Pore I and Pore II). Pore I corresponds to the proposed model of Suzuki et al. [30], with a “face-to-face” arrangement of cldns. The Pore II structural model represents a “back-to-back” arrangement of cldns that is mediated through interactions between the two transmembrane helices TM2 and TM3. This dimer model, in which one cldn monomer is rotated ~180° from its partner, features the same pore-lining residues from Pore I, albeit with the positively charged residues K65 and K48 located at the pore entrances [45]. Subsequent studies identified higher-order aggregates of cldn-1, -2, -4, and -19 in CG simulations including dimeric and trimeric interfaces [46]. The dimeric interface of Suzuki et al. [30] was again one of the dominant interfaces and was used in constructing an all-atom model of cldn-2 pores [46]. The authors chose to test cldn-2 only in the context of Pore I, and thus, there is no assessment or direct comparison of the stability of the two models for cldn-2. The pore diameter and pore-lining residues were overall consistent with previous studies, with D65 and Q63 facing the ion transport pathway. 

Berselli et al. investigated the functional properties of cldn-4 in the context of these two pore models (Pore I and Pore II) [47]. A free energy calculation of ion transport through cldn-4 pores indicated the Pore I forms an anion-selective channel with an attractive well at the position of K65 inside the pore. In contrast, due to drastic changes in the pore-lining residues, Pore II is not permeable to cations or anions with an energy barrier of ~5 kcal/mol. These results indicate that only the Pore I structure (Suzuki model) is consistent with the anionic selectivity of cldn-4 paracellular channels. It should be noted that in this study, the Cα atoms of the extracellular domains in the Pore II arrangement were restrained to ensure the stability of the structure within the membrane. 

In a similar study, Berselli et al. examined Pore I and Pore II models for cldn-5, a barrier-forming cldn in endothelial cells of the blood–brain barrier [45]. The results of their all-atom molecular dynamics simulations showed that the Pore I arrangement is structurally more stable. In addition, by calculating the free energy profile of the ions inside the pore, they concluded that in both arrangements, cations or anions face a relatively high energy barrier for passing through cldn-5, consistent with cldn-5′s known barrier function [45]. 

It was recently discovered that a single-point mutation in cldn-5, G60R, converts this barrier-forming cldn into an anion-selective channel [48]. Berselli et al. examined the ion transport properties of the G60R mutant of cldn-5 in the context of Pore I and Pore II models. G60 is located on ECS1, and its mutation to R (G60R) does not alter tight junction formation in MDCKII cells [48]. However, its mutation affects the cldn-5 barrier function by inducing high Cl^−^ permeability and low Na^+^ permeability through cldn-5. Berselli et al. used umbrella sampling molecular dynamics simulations to calculate the free energy profiles of Na^+^ and Cl^−^ ions within the mutant pore. Their results indicate that only Pore I (Suzuki model) is consistent with the anion-selective properties of this mutant. The mutation reduced the energy barrier for Cl^−^ translocation inside Pore I from approximately +1 kcal/mol to −2.5 kcal/mol, providing an attractive minimum for Cl^−^ ions, and increased the energy barrier for Na^+^ ions from approximately +3 kcal/mol to +10 kcal/mol, consistent with an anion selective channel. In Pore II, the mutation did not affect the free energy profiles of Na^+^ and Cl^−^ ions in cldn-5 [45], indicating that only Pore I is consistent with the experimental data on cldn-5 [49]. 

Berselli et al. tested the effect of two additional mutations, namely Q57D and Q63D, in cldn-5. They limited these studies to the Pore I arrangement and calculated the free energy profile for ion translocation inside the pore. These two residues were selected because they are close to G60 and are within the constriction zone in the middle of the pore. Q57 is highly conserved among classic cldns, except for cldn-2, cldn-10b, cldn-14, and cldn-15, where it is replaced by an aspartate (D) in cldn-10b and -15 or a histidine (H) in clnd-2 or -14. Q63 is also highly conserved among cldns, and in cldn-15 and cldn-10b, it is replaced by an asparagine (N) [49]. A single mutation of Q57D in cldn-5 was sufficient to induce cation selectivity in the channel, with a barrier very similar to that of cldn-15. Q63D, on the other, was still not permeable to Cl^−^ ions but had a slight permeability to Na^+^ [49].

In a recent investigation, Hempel et al., used a combination of computational and experimental techniques to assess possible configurations of cldn-3 and cldn-10b monomers in TJs [50]. They concluded that the back-to-back assembly of cldns, corresponding to the Pore II model [43], is not relevant for the *cis* polymerization of cldn-3 or cldn-10b within the TJ. However, the face-to-face interface as proposed by Suzuki et al. [30], corresponding to the Pore I arrangement (the Suzuki model), is consistent with cldn polymerization within the membrane. These results put previous structural modeling of cldns into perspective; a mere observation of the dimeric interface in docking or CG simulations does not indicate the relevance of that dimer interface in the *cis* polymerization of cldns within the TJ. 

For cldn strands composed of more than one cldn subtype (heterotypic), the connection between cis interfaces and the TJ strand architecture remains largely unknown. Raya-Sandino et al. examined the heterogeneous assemblies of clnd-23, cldn-3, and cldn-4 through docking and identified possible dimeric interfaces that were energetically favorable [51]. Molecular dynamics simulations of heterostructures in the context of the Suzuki model led to characterizations of the pore radii and electrostatic interactions of the pore surface. In particular, heterostructures consisting of cldn-23 and cldn-3 or cldn-4 had significantly reduced pore compared to homo-typical structures. The minimum diameter of the heterostructures made of cldn-23/cldn-4 or cldn-23/cldn-3 was approximately 0.6–0.8 Å, indicating an almost impermeable pore consistent with the observation that adding cldn-23 to TJs reduces its permeability. While these models and simulations are informative in providing insights on the pore-lining surfaces of heterostructures, they do not verify the potential *cis* or *trans* interactions observed in these assemblies. This is because the simulations were carried out with positional restraints on the transmembrane helices of the tetramers to enforce the assembly and prevent the destabilization of the tetramer. 

In summary, the collective findings from various experimental and computational studies indicate a consensus regarding the validity and practical applicability of the double-row arrangement of Suzuki et al. [30] in cldn polymerization. CG simulations have been instrumental in providing the first glimpse of cldn assembly in lipid membranes. However, CG simulations are inherently limited in reproducing intermolecular interactions. CG simulations are known to lead to observations that are governed by the shape of the proteins and the hydrophobic/hydrophilic interactions between system components rather than by interatomic interactions. As such, rigorous analyses of the CG results alongside all-atom control simulations are needed to verify the results. 

## 4. A Mechanism for Lateral Flexibility at *cis* Interfaces

To examine the dynamic morphology of cldn strands and their mechanical properties, it is essential to model intermolecular interactions at an atomic resolution. Fuladi et al. simulated double stands of cldn-15 in two parallel lipid membranes ranging from 27 nm to 225 nm in size [52]. These simulations were carried out at a hybrid resolution, where the protein was modeled atomically, but lipid and water molecules were coarse grained (PACE forcefield) [53,54,55]. The strands consisted of 36 to 300 cldn-15 monomers arranged linearly according to the double-row model of Suzuki et al. [30]. The initial conformation of the monomers, however, was taken from a refined model of the cldn-15 pores in lipid membranes [33]. The hybrid-resolution simulations not only reduce the computational cost required to reach the long-timescales needed to study the dynamics of the strands, but also facilitate the dynamics of the proteins in the membrane. The hybrid-resolution simulations reproduced the all-atom results, with properly hydrated paracellular pores within the cldn-15 channels [52]. The head-to-head *trans* and *cis* interactions were well maintained. However, the dynamic curvature of the strand was more apparent in the hybrid-resolution models, with fluctuations in the local curvature of the strands.

Fuladi et al. [52,56] calculated the persistence length of the cldn-15 strands from the thermal fluctuation of the strands in the membranes. The persistence length (*l_p_*) is an indication of the strand rigidity and is directly related to the bending modulus (κ_B_) of the strands and the temperature (T). The lateral flexibility of the strands was calculated from thermal fluctuations of the system in equilibrium simulations, indicating a persistence length of *l_p_* = 137 ± 47 nm over all strands [52]. This is in close agreement with the experimentally measured persistence length of cldn-15 by Zhao et al. (191 ± 184 nm) [39], indicating that the dynamic of the strands captures the overall shape of the TJ.

Zhao et al. estimated the persistence length of cldn-15 by analyzing the curvature of strands from freeze-fracture electron microscopy images of over 50 strands and correlating the average curvature and its fluctuations to the persistence length at room temperature [39]. The two methods are theoretically equivalent; however, to estimate the average curvature, one needs multiple samples (or snapshots) of the strands spanning all possible curvatures. Most importantly, the strands must be either longer or of the same order of the persistence length: in this case, ~200 nm. However, the bending rigidity (κ_B_) of the strands can be calculated directly from equilibrium simulations of the strands at any length, as long as the simulations are long enough to capture local fluctuations in the curvature. As demonstrated by Fuladi et al., the persistence length obtained from 27 nm strands with 36 monomers is comparable to those obtained from 225 nm strands with 300 monomers [52]. 

The lateral flexibility of cldn-15 strands in the membrane stems from flexible arrangement of cldn-15 monomers with respect to each other. Upon bending of the strands, adjacent cldns in a row rotate with respect to each other and form new contacts to adapt to the local curvature. Microsecond-long simulations of the strands at room temperature indicate that the relative orientation angle of neighboring monomers ranges from −50° to +50°, with a probability distribution that is centered and peaks around 0°. In fact, 68% of the time, the orientation angles were between −26° to +26°. It must be noted that these statistics could only be obtained from the 300 cldn-15 strands and from μs long trajectories. 

**Figure 2:**
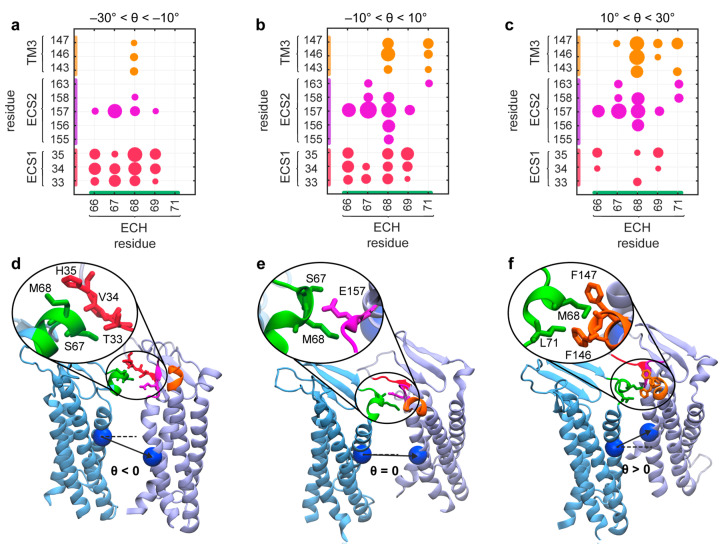
Multiple side-by-side *cis* interaction interfaces provide the flexibility of cldn-15 strands. (**a**–**c**) Contact maps of the side-by-side *cis* interactions between cldn pairs grouped into three clusters according to their relative orientations. The size of the circle denotes the probability of a pairwise interaction, and the colors correspond to the interacting region. The analysis corresponds to the thermal fluctuation of a 300 cldn strand in two parallel lipid membranes. (**d**–**f**) The snapshots of dominant interactions at each cluster are shown for cldn pairs corresponding to (**d**) negative, (**e**) zero, and (**f**) positive orientation angles. The interacting regions are colored green for ECH, red for ECS1, magenta for ECS2, and orange for TM3. The figure is reproduced from [52] with permission.

An examination of the contact maps between cldns and their frequency highlights the main residues involved in the side-by-side *cis* interactions (Figure 2). The analysis uncovered three sets of side-by-side *cis* interactions between cldns involving the extracellular helix (ECH) and specific regions, including ECS2/TM4, TM3, and ECS1. The extracellular helix ECH serves as a pivot point for the rotation of cldn monomers around a centrally positioned ECH–ESC2 interface. Positive curvatures lead to reduced contact between ECH and ECS1, with an increased interaction between ECH and TM3, while negative curvatures enhance ECH–ECS1 interactions. Monomers span this range of motion without disrupting the *cis* interaction between them or compromising the integrity of the strands. Notably, an alternative interface proposed by Zhao et al. corresponding to a 17° rotation between the monomers falls within this range. 

**Figure 3:**
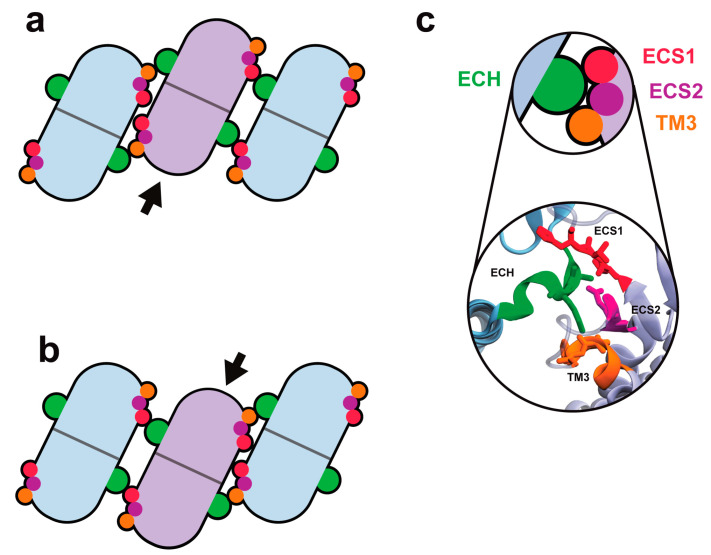
(**a**,**b**) Tetrameric cldn channels slide with respect to each other to respond to local fluctuations in the strand curvature. The lateral flexibility of the strand originates from the robustness of side-by-side *cis* interactions via the three interacting regions highlighted in (**c**). The figure is reproduced from [52] with permission.

These simulations [54] not only corroborate the flexibility of the strands, but also suggest a molecular mechanism for cldn-15 assembly which explains the lateral flexibility of the strands without a loss of function or pore leakage. Simulations show that as the strands curve due to thermal fluctuations or external forces, side-by-side *cis* interactions adapt and allow for the slight displacement of monomers with respect to each other. This displacement could potentially break the tetrameric structure of cldn channels. However, the face-to-face *cis* and head-to-head *trans* interactions remain in place despite fluctuations to the strand. Simulations reveal that upon bending of the strand, tetrameric ion channels move as a single unit and slide with respect to each other to accommodate the curvature [52]. The ion channels remain intact during this process, as summarized in Figure 3. 

In fact, the lateral flexibility of the strands is due to an interplay of three sets of interactions with variable strengths: the side-by-side *cis* interactions, which are flexible and accommodate a range of motion between neighboring monomers; the face-to-face *cis* interactions between β-sheets, which are flexible and slide with respect to each other to maintain the β-barrel core of cldn channels through a motion that is prevalent in mechanical proteins; and the *trans* interactions between the extracellular loops of cldns within the two lipid membranes, which are the most resilient. 

Remarkably, this working hypothesis of sliding monomers is supported by hydrogen bond analyses of face-to-face *cis* interactions in MD simulations of membrane-embedded cldn-15 [33,34] and cldn-10b [38] in octameric interlocked-barrel conformations, with an average of ~two hydrogen bonds between face-to-face *cis* interfaces in these simulations. A recent crystallographic structure model of cldn-3 and two of its mutants, P134A and P134G, suggests that *cis* interactions between cldns are indirectly affected by a single mutation at position 134 on the third transmembrane helix (TM3) of cldn-3 [15]. In cldns, this position is mainly occupied by alanine (as in cldn-15) or proline (as in cldn-3). The protein structures of cldn-3 and cldn-15 are very similar, but the TJ strands formed by cldn-3 have a distinct morphology with sparsely distributed, almost linear strands. In contrast, TJ strands formed by two cldn-3 mutants (P134A and P134G) are more flexible, with several hairpin curves similar to those formed by cldn-15 [15].

**Figure 4:**
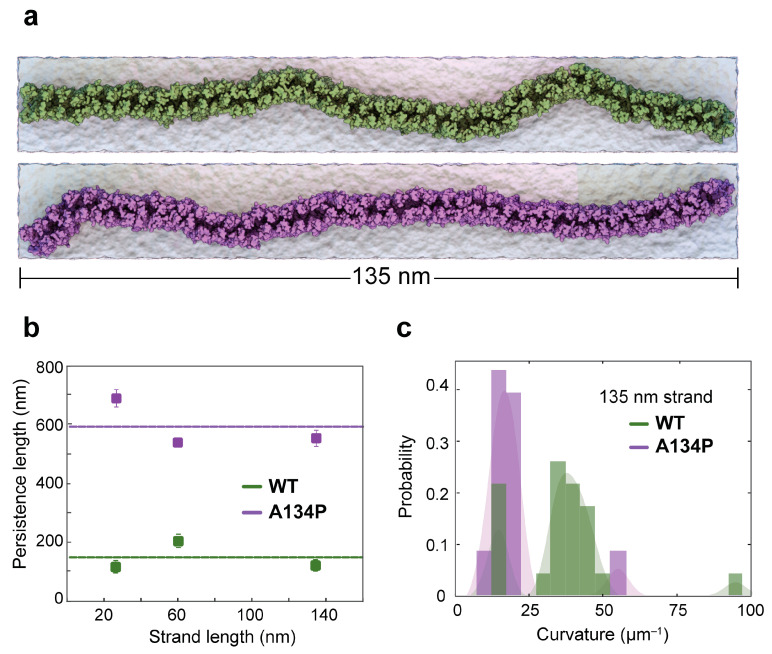
TM3 bending restricts cldn-15 strand flexibility. (**a**) Equilibrated configurations of 135 nm WT and A134P strands simulated for 1 μs in two parallel lipid membranes. Snapshots show the “curvy” morphology of the WT cldn-15 strand in comparison to the more “straight” shapes of the A134P mutant strands. (**b**) The strand persistence length is calculated for the WT and A134P cldn-15 strands. The horizontal lines show the average persistence lengths for WT and A134P mutant strands. (**c**) The distribution of the local curvature along the length of the longest simulated strand (135 nm) is calculated for WT and A134P strands. The figure is modified with permission from [56].

Fuladi et al. studied the effect of this mutation in cldn-15 [56]. Using a combination of all-atom and hybrid-resolution simulations, they showed that a single mutation of A134P in cldn-15 increases the persistence length (*l_p_*) of cldn-15 strands by a factor of 4 (590 ± 79 nm vs. 137 ± 47 nm for cldn-15 WT) (Figure 4). Analyses of *cis* interactions between cldns indicate that the mutation does not alter the intermolecular contacts between cldns. However, the dynamics and relative occupancies of interfacial contacts are significantly affected. The A134P mutation in cldn-15 introduced a kink in the third transmembrane helix (TM3) of the protein, similar to the one observed in cldn-3’s crystal structure. The kink in TM3 skews the rotational flexibility of the cldns in the strand, favoring the interaction between ECH and TM3 (corresponding to the positive angles in Figure 2c). This asymmetry would generally lead to higher curvatures in the strands; however, in the context of the anti-parallel, double-row strands, the asymmetric movement reduces the lateral flexibility of the strands and increases the persistence length of the mutant. These results indicate an indirect role of TM helices, not necessarily in cldn assembly, but in modulating their dynamics. 

## 5. Evaluation of Computational Methods and Models

In the previous sections, we reviewed the latest computational studies on cldn assembly into paracellular ion channels and explored their functional properties. A diverse array of computational methods ranging from protein–protein docking, all-atom molecular dynamics simulations, coarse-grained simulations, and hybrid-resolution simulations have been employed to investigate the structure and function of cldn channels. However, not all methods are equally reliable. Each method has its own limitations, which sometimes lead to contradictory results. Here, we briefly discuss the shortcomings and advantages of each method and the limitations of the resulting models.

### 5.1. Protein–Protein Docking

Protein–protein docking is a computational method for predicting the interfacial surfaces of protein complexes. It is particularly useful when the experimental determination of the protein complex is difficult [57]. However, in contrast to ligand–protein docking studies, the success rate of protein–protein docking is limited. This is due to the complexity of protein–protein interactions and the presence of additional constraints such as the presence of lipid membranes, which limits the protein orientation. In addition, due to the intrinsic susceptibility of the docking methods to false-positive results, the accuracy of the protein–protein interfaces suggested by the docking method cannot be confidently asserted and requires alternative methods and experimental validation [57,58]. 

### 5.2. All-Atom Molecular Dynamics Simulation

All-atom molecular dynamics simulations provide the resolution needed and a realistic representation of the forces and timescales involved to reproduce the finely tuned network of interactions between proteins, lipids, and water molecules [59]. However, the timescales achievable for all-atom molecular dynamics barely reach milliseconds, which is generally not enough to provide a mechanistic insight into protein assemblies [60]. However, within sub-millisecond timescales, all-atom molecular dynamics simulations can capture the dynamics of assembly and disassembly of protein–lipid complexes. An example is the molecular dynamics simulations of the SNARE-mediated presynaptic fusion complex, which shed light on the dynamics of vesicle–plasma membrane fusion, agreeing with structural experimental data [61]. Another example is the simulations of a prototypical adhesin and fibrinogen complex, where the simulations successfully reproduced the mechanical properties of the system, consistent with single-molecule force-spectroscopy [62]. Microsecond-long simulations of the Piezo channel have captured membrane deformation coupled to conformational changes in the channel [63]. 

While all-atom simulations are limited in reaching the millisecond timescales required for protein assembly, they are powerful tools to study the transport mechanisms of ions and small molecules through channels. Simulations of ion transport through the refined models of cldn-15 (Suzuki model) verified the function of the cldn-15 pores as cation-selective ion channels [33,34,35]. Different approaches were employed to study ion transport [34,35]; Samanta et al. simulated ion transport by applying an electric field across cldn pores [33]. The electric field drives the transport of the ions through the channel. However, to determine the ion permeabilities and channel selectivity, it is necessary to run the simulations at a constant voltage. The application of an electric field across a dielectric barrier, like the lipid bilayer surrounded by water, is shown to produce a voltage drop across the membrane that is proportional to the length of the simulation box [64]. Cldn channels are not situated in the membrane, and thus, this method is not always applicable to them. To generate a constant voltage drop across cldn channels, one needs to seal the paracellular space within the two membranes by allowing ions and water to pass only through cldn pores. This was achieved in the work of Samanta et al., by selecting a unit cell in which cldn channels form a strand that continues across the boundaries of the unit cell, forming a continuous strand with punctured holes, thus enabling them to calculate the conductivity of the cldn-15 pore and its mutants to cations and anions from current–voltage relationships [33]. Although applying a non-physiologically high electric field to the simulation box may cause discrepancies in the electrostatic environment found in biological systems and introduce non-linearities into the system, this strategy has been successfully implemented in studying the ion permeation of proteins [65,66,67,68,69,70].

Alberini et al. adopted a different strategy [35]. Their simulation system consisted of single or double cldn-15 pores between two membranes surrounded by water and ions. The water-filled region around the cldn-15 pores in this case would provide a low-resistance pathway for the ions to flow, resulting in leak pathways parallel to the cldn-15 pore. A constant electric field, in this case, would not lead to a constant voltage drop across the pores, and thus cannot be used to calculate ionic currents. As such, to assess the functional properties of the cldn-15 model, Alberini et al. calculated the potential of mean force for the passage of ions through the pore. By calculating the free energy barrier for the passage of ions, they determined that cldn-15 is cation selective, with a relatively high free energy barrier for anions like Cl^−^. The authors have used the same approach to calculate the energy barrier for the passage of ions in cldn-4 and cldn-5 and its mutants [45,47,49].

Although limited by the accuracies of the available interatomic forcefields, these simulations provided valuable insight into ion permeation and the selectivity mechanisms of cldn channels and helped with the design of new mutants with a modified charged selectivity [33,49].

### 5.3. Coarse-Grained Molecular Dynamics Simulations

Coarse-grained molecular dynamics simulations can access longer timescales that are required to study protein complexes. However, they lack the atomic resolution needed for protein assemblies. They are limited in capturing protein conformational changes or maintaining the secondary structure of the proteins. By reducing the number of particles in the system, coarse-grained models enable simulations of larger structures for longer times at the expense of lost atomic interactions. This approach has been successful in modeling phenomena or assemblies that are governed by hydrophobic/hydrophilic interactions, such as vesicle formation or fusion, or are controlled by the overall three-dimensional shape of the protein and are less sensitive to inter-protein atomic interactions.

In the case of cldns, where the formation of strands depends on a delicate balance between *cis* and *trans* interactions, coarse-grained simulations lack the precision to accurately predict the structural assemblies of cldn subtypes and distinguish them. This challenge is more pronounced in the absence of experimentally derived structures for different cldn subtypes. The majority of coarse-grained studies of cldn assembly directly use the structure of cldn-15 [29] or employ it as the foundation for homology models of other cldn subtypes. Thus, it is not surprising that none of the models predicted using the coarse-grained simulation, except for the Suzuki model, were validated experimentally.

### 5.4. Hybrid-Resolution Molecular Dynamics Simulations

Hybrid-resolution methods strike a balance between atomic detail and computational cost, providing the right resolution to study protein assemblies and their dynamics. This is achieved by reducing the degrees of freedom in the system, allowing for simulations of larger and more complex systems. The hybrid-resolution model PACE [54,55] was used successfully to reproduce the persistence length of cldn-15 strands and its mutants without any loss in the resolution when modeling protein–protein interactions [52,56]. This is due to the fact that the persistence length is a time-independent parameter and is not affected by the altered dynamics of the coarse-grained lipid molecules. Hybrid-resolution simulations were also employed to study Piezo membrane systems to model the membrane topology, a key factor for inducing Piezo channel gating [63,71]. The PACE forcefield was also used to study amyloid aggregation by simulating the interaction of α-synuclein with complex lipid bilayers and its structural transition during membrane insertion [72,73].

It has to be noted that coarse-grained simulations inherently alter the timescales of the events, and thus, time-dependent parameters such as transition rates cannot be estimated from coarse-grained simulations. Similarly, time-dependent parameters cannot be extracted from hybrid-resolution simulations. However, the persistence length can be calculated from the bending modulus of cldn strands, which is a time-independent parameter and is not affected by the modified dynamics of the system [52,56].

However, hybrid-resolution simulations are limited in their capabilities and cannot be used to study the time dependency of biological processes, such as diffusion or association and dissociation rates. Furthermore, coarse-grained solvents and ions limit the accuracy of hybrid-resolution models for ion transport studies. The introduction of other compounds, such as ligands that interact with the protein, e.g., by binding to the ion transport pore, is not trivial and requires the development of additional forcefield parameters for the ligand with other components of the system, such as coarse-grained water or lipid molecules.

### 5.5. Additional Limitations

In addition to the intrinsic limitations of the methodologies discussed above, the computational studies reviewed here often adopt a simplified model of cldns and their assembly in tight junctions. Complex lipid compositions are not always considered, and the role of lipid membranes is generally reduced to a homogeneous environment without any specific protein–lipid interactions. Furthermore, post-translational modifications, such as palmitoylation and phosphorylation, as well as their potential impact on cldn interactions with lipid membranes, have been commonly disregarded.

Tight junctions are associated with lipid raft-like membrane microdomains enriched with cholesterol, sphingomyelin, and long-chain fatty acids, which play a role in claudin localization and tight junction formation [74,75,76]. Irudayanathan et al. explored seven different lipid compositions with variable hydrophobic thicknesses, performing 10 µs long coarse-grained simulations of cldn-5 polymerization in mixed lipid compositions, including POPC or DPPC with cholesterol and ceramides [42]. These studies suggest that the presence of cholesterol discourages cldn-5 aggregation into strands and affects the preferential arrangement of cldn-5 into dimers.

Palmitoylation, i.e., the addition of palmitic acid to cysteine residues on the TM2 and TM3 helices of cldns, is shown to affect cldn localization [77,78,79,80]. Palmitoylated claudins have been found to interact with scaffolding proteins (ZO) and cholesterol to enable tight junction formation, challenging the canonical understanding that protein–protein interactions between cldns and ZO proteins drive TJ assembly [75]. While some studies explored the role of palmitoylation on cldn-5 assembly in coarse-grained simulations [79], the impact of palmitoylation on cldn strand assembly is still unclear. Atomic simulations of cldns embedded in biologically relevant membranes are needed to refine our understanding of cldn–lipid interactions and their impact on cldn strand architecture.

## 6. Conclusions and Outlook

This review provides an overview of the recent computational studies on cldn assembly and strand formation. Computational studies have reached a consensus on the most probable *cis* interactions between cldns. These models explain the nature of ion selectivity in cldn-15, cldn-4, cldn-2, and cldn-10b, as well as the barrier function of cldn-5. In addition, simulations of cldn strands with up to 300 monomers describe the lateral flexibility of cldn-15 strands in agreement with experiments. In these models, cldns assemble into an interconnected strand of tetrameric ion channels that move collectively, adapting to the local curvature under mechanical forces. Flexible *cis* interactions dynamically fluctuate around a centrally positioned interface, initially identified in crystallographic studies. Face-to-face *cis* and head-to-head *trans* interactions sustain the tetrameric structure of ion channels during strand bending and shaping, preserving the barrier function.

In summary, these studies provide a molecular mechanism for cldn strand assembly that is consistent with the lateral flexibility of the strands in the membrane and shed light on the cooperative natures of *cis* and *trans* interactions among cldns in linear, flexible strands. These studies set the groundwork for future computational and experimental studies on the roles of key cldn amino acids in the precise architecture and organization of TJs. Further studies of more complex systems are needed to determine the roles of other TJ-associated proteins in TJ branch formation and their dynamics.

## Data Availability

No new data were created or analyzed in this study. Data sharing is not applicable to this article.

## Abbreviations

The following abbreviations are used in this manuscript:

Cldn	claudin
MD	molecular dynamics
CG	coarse grained
TJ	tight junction
TM	transmembrane
ECS	extracellular segment
ECH	extracellular helix
